# The adult outcome of childhood quasi‐autism arising following extreme institutional deprivation

**DOI:** 10.1111/jcpp.13767

**Published:** 2023-02-13

**Authors:** Maria Rodriguez‐Perez, Mark Kennedy, Edward D. Barker, Jana Kreppner, Mireia Solerdelcoll, Edmund J.S. Sonuga‐Barke

**Affiliations:** ^1^ Department of Child and Adolescent Psychiatry, Institute of Psychiatry, Psychology and Neuroscience King's College London London UK; ^2^ Department of Psychology, Institute of Psychiatry, Psychology and Neuroscience King's College London London UK; ^3^ Centre for Innovation in Mental Health, School of Psychology University of Southampton Southampton UK; ^4^ Department of Medicine University of Barcelona Barcelona Spain; ^5^ Department of Child and Adolescent Psychiatry Aarhus University Aarhus Denmark

**Keywords:** Autism, quasi‐autism, institutional deprivation, early adversity, Romanian adoptees, longitudinal

## Abstract

**Background:**

Rutter and colleagues' seminal observation that extended early life exposure to extreme institutional deprivation can result in what he termed quasi‐autism (QA), informed both our understanding of the effects of adversity on development and the nature of autism. Here we provide the first detailed analysis of the adult outcomes of the group of institutionally deprived‐then‐adopted children identified as displaying QA.

**Methods:**

Twenty‐six adult adoptees identified with QA in childhood (*Childhood QA+*) were compared to 75 adoptees who experienced extended institutional deprivation (>6 months) but no QA (*Childhood QA*−), and 116 adoptees exposed to *Low/No institutional deprivation*. The outcomes were child‐to‐adult developmental trajectories of neuro‐developmental symptoms (autism, attention‐deficit/hyperactivity disorder (ADHD), disinhibited social engagement (DSE) and cognitive impairment), adult functioning, life satisfaction and mental health.

**Results:**

*Childhood QA+* was associated with elevated and persistent trajectories of broad‐based autism‐related difficulties, ADHD and DSE symptoms and low IQ, as well as adult mental health difficulties and functional impairment, including high rates of low educational attainment and unemployment. Life satisfaction and self‐esteem were unaffected. Autism‐related communication problems, in particular, predicted negative adult outcomes. *Childhood QA+* was still associated with poor outcomes even when ADHD, DSE and IQ were controlled.

**Conclusions:**

Early and time‐limited institutional deprivation has a critical impact on adult functioning, in part via its association with an early established and persistent variant of autism, especially related to communication difficulties. Apparent similarities and differences to non‐deprivation related autism are discussed.

## Introduction

Autism is a childhood‐onset neurodevelopmental condition characterised by difficulties with social interactions, communications and repetitive and stereotyped behaviours (American Psychiatric Association, 2013). It has a strong genetic component according to twin (Tick, Bolton, Happé, Rutter, & Rijsdijk, 2016) and molecular genetic studies (Glessner, Connolly, & Hakonarson, 2014). Pre‐conception, prenatal and perinatal environmental factors may also be important (Gardener, Spiegelman, & Buka, 2011; Kerin et al., 2018; Kolevzon, Gross, & Reichenberg, 2007; Ornoy, Weinstein‐Fudim, & Ergaz, 2015; Wu et al., 2017). Common, post‐natal social environmental factors, for instance those affecting the quality of the parent–child relationship, have been ruled out as a primary cause of autism (Rimland, 1985), although complex and subtle interactions with genetic factors may occur (Mandy & Lai, 2016). Complicating the picture, Rutter et al. (1999) reported exceptionally high rates of marked autistic features in children adopted into UK families after they spent extended periods (up to 43 months) in brutally depriving Romanian institutions of the 1980s. This raised the possibility that exposure to extreme and extended negative social experiences, early in life, could lead to autism.

Based on a combination of clinical observations and systematic assessments using validated instruments (Rutter et al., 1999, 2007), 20 children were deemed to meet full diagnostic criteria while an additional eight met clinical thresholds on screening questionnaires (overall 16% of the sample). All had experienced more than 6 months of deprivation. Initial reports suggested that childhood deprivation‐related autism differed from idiopathic autism in several ways – including an unusual degree of social approach, a relative lack of communication problems, a high degree of intense circumscribed interests and/or abnormal preoccupations, as well as a weaker association with male sex and less persistence between ages 4 and 6 years. Rutter coined the term ‘*quasi‐autism*’ (QA) to describe this presentation (Rutter et al., 1999). More generally, QA formed part of a highly distinctive, yet heterogeneous and only partially overlapping, set of what were termed deprivation‐specific problems (DSPs) (Rutter, Sonuga‐Barke, & Castle, 2010) ‐ attention‐deficit/hyperactivity disorder (ADHD), symptoms of disinhibited social engagement disorder (DSE) and cognitive impairment (i.e. IQ < 80).

Follow‐up studies into adolescence  (Kreppner et al., 2010; Rutter et al., 2007) demonstrated the strong persistence of all DSPs symptoms from age 6 years, including autism symptoms (Kreppner et al., 2010; Rutter et al., 2007) – despite extended periods of good quality adoptive care for nearly all adoptees. More recently, the same pattern of symptom persistence was observed into early adulthood for autism and other DSPs (Sonuga‐Barke et al., 2017). In terms of autism symptoms, these recent analyses left many scientific questions of clinical significance unanswered. First, it reported only a single measure of aggregated symptoms across the different autism domains (communication, social reciprocal interactions, and repetitive and stereotyped behaviours) and, therefore, was unable to ask questions about the deprivation‐related autism symptom profile or which aspects of it drove developmental outcomes. Second, it focused on comparing groups of deprived and non‐deprived individuals as a whole and so was unable to conclude anything about what distinguished adoptees who had experienced extended deprivation with and without previously identified clinically significant autistic symptoms (i.e. QA). Third, there was not enough space to give a sufficiently fine‐grained analysis of the impact of deprivation‐related autism on different aspects of adult functioning to help inform clinical decision‐making. The current paper aims to address these outstanding issues to establish the developmental significance and clinical status of the different components of childhood‐identified QA vis‐à‐vis other deprivation specific problems, on the one hand, and idiopathic autism on the other.

To do this, we compared young adults who had experienced extended institutional deprivation (>6 months) and were identified in childhood as having QA (*Childhood QA+*) with those who experienced the same level of deprivation but were not identified as having QA (*Childhood QA−*) with a combined group of Romanian adoptees who experienced less than 6 months of institutional deprivation and UK adoptees who experienced no deprivation, both without QA (*Low/No Deprivation*). These group designations have been used in many ERA analyses previously because: (a) there have been equally low levels of DSPs in non‐deprived UK adoptees and Romanian adoptees who experienced <6 months deprivation for all analyses at all follow‐up ages, and (b) there was a striking step increase in DSPs rates for those who experienced more than 6 months of deprivation with little further increase in risk associated with each additional month of deprivation (see Golm et al., 2020; Kennedy et al., 2016, 2017; Sonuga‐Barke et al., 2017). We addressed the following research questions:

1. Compared to the *Low/No Deprivation* and the *Childhood QA−* groups, does the *Childhood QA+* group:
  - display persisting symptoms in all three autism core domains (social reciprocity, communication, and repetitive and stereotyped behaviours) across adolescence through to adulthood?
  - display elevated trajectories of other DSPs into adulthood (i.e. ADHD, DSE and cognitive impairment)?
  - experience poor mental health, impaired daily functioning, reduced life satisfaction and self‐esteem in adulthood?
2. Is the association between childhood QA and adult functioning and mental health independent of the effects of other deprivation‐specific neurodevelopmental problems – DSE, ADHD and cognitive impairment expressed from childhood through to adolescence?
3. Which autism symptom domain (social reciprocity, communication and/or repetitive and stereotyped behaviours) is most strongly associated with the negative impact of QA on adult outcomes (i.e. mental health and functional impairment)?

## Methods

### Participants

One hundred and sixty‐five Romanian adoptees and 52 comparisons UK adoptees with no history of institutional deprivation, and their families, entered the ERA study in the mid‐1990s. Romanian adoptees typically entered institutions in the first few months of life and experienced between 1 and 43 months of institutional deprivation. For the current analysis, as in previous studies, Romanian adoptees with <6 months of institutional deprivation were combined with a group of UK adoptees, also adopted before 6 months, into a *Low/No Deprivation* group. This is because prior analyses (e.g. Kreppner et al., 2007; Sonuga‐Barke et al., 2017) have shown that elevated risk of difficulties is almost entirely restricted to Romanian adoptees experiencing over 6 months of institutional deprivation so that the risk in the Romanian and UK adoptees adopted before 6 months was equivalently low (Sonuga‐Barke et al., 2017). We compared 26 adoptees with childhood QA (*Childhood QA+*) with 75 adoptees with over 6 months of exposure to institutional deprivation but no childhood QA (*Childhood QA−*), and 116 *Low/No Deprivation* group members (64 Romanian adoptees with <6 months of institutional deprivation and 52 UK adoptees without deprivation). The *Childhood QA+* group consisted of 20 individuals who were identified with marked autistic feature based on the clinical assessment at both/either 4/6 and 12 years (ADI; Lord, Rutter, & Le Couteur, 1994; ADOS; Gotham, Risi, Pickles, & Lord, 2006). A further group of eight children met clinical cut‐offs on the Social Communication Questionnaire only (i.e. SCQ total score > 14; Rutter, Bailey, & Lord, 2003). Only 6 of these were included in the analyses as 2 were excluded because of the presence of specific biological risk factors (e.g. foetal alcohol syndrome). There were no significant differences between the 20 individuals who met the ADOS/ADI criteria and the six who just met the SCQ thresholds (see Appendix S1 – Table S1).

ERA study retention was high up to age 15 years (data available from 90% of the sample) but decreased into adulthood (data missing for 25% of the sample). There was no evidence of selective attrition comparing those dropping out prior to the young adult assessment and those who remained in the study (data available on requests; see Sonuga‐Barke et al., 2017). Ethical approval was received from University of Southampton Research, and King's College London, Ethics Committees.

### Measures

Measures were collected at age 4, 6, 11 and 15 years and in young adulthood (average age 23 years). All measures used have acceptable psychometric properties (details available on request).

### Background characteristics

Adoptees' sex, date of birth and duration of institutional deprivation was obtained from Romanian records taken at the time of entry into the United Kingdom. Social‐economic status (SES) was based on adoptive parents' occupation at the age 15 follow‐up (Rutter & Sonuga‐Barke, 2010). Families were divided into high (i.e. skilled, managerial/technical and professional occupations) and low (i.e. manual and unskilled occupations) status (General Registrar Office, 1971). Parental marital status derived from young adult reports was coded marriage intact = 1, divorced/separated/widowed = 0.

### Deprivation‐specific problems assessed at all follow‐ups

#### Autism symptoms

The parent reported SCQ (Rutter et al., 2003) was used to assess autism symptoms across the three core domains – social reciprocity, communication, and repetitive and stereotyped behaviours. For our analysis we selected five items from each domain which were developmentally appropriate across all assessment waves. These can be seen in Appendix S2 – Table S2 and S3 (see Sonuga‐Barke et al., 2017 for the rationale for this approach). Items were rated 0 for ‘absent’ or 1 for ‘present’.

#### ADHD symptoms

In order to ensure developmental consistency across assessment waves, hyperactivity, sustained attention and distractibility were measured using equivalent items from the Revised Rutter Scale (Elander & Rutter, 1996) at ages 6 and 11, the Strengths and Difficulties Questionnaire (SDQ; Goodman, 1997) at age 15, and the Conners Comprehensive Behaviour Rating Scale (CBRS; Conners, Pitkanen, & Rzepa, 2011) in young adulthood. For the Revised Rutter Scale and the SDQ, a symptom was deemed endorsed when a rating of 2 (‘certainly applies’) was made (0–2 scales). The equivalent rating (‘often/very often’, rating of 2 or 3) was found in the CBRS (0–3 scale; see Sonuga‐Barke et al., 2017 for rationale).

#### DSE

At all ages this was based on researcher ratings of parents' responses to three interview questions. These tapped the constructs of being *too friendly*, *showing inappropriate intrusiveness* and *being unaware of social boundaries*. A rating of ‘definite evidence of disinhibition’ represented a positive endorsement.

#### IQ

The McCarthy Scales of Children's Abilities General Cognitive Index (McCarthy, 1972) was used at age 6, the short form of the Wechsler Intelligence Scale for Children (WISC; Wechsler, 1974; i.e. block design, object assembly, vocabulary and similarities) at ages 11 and 15 years. The block design and vocabulary subscales from the short‐form Wechsler Abbreviated Scale of Intelligence (WASI) (Wechsler, 1999) were assessed in adulthood.

### Mental health and wellbeing in adulthood

#### Psychopathology

Generalised anxiety, major depression, oppositional defiant disorder, manic episode, social phobia, conduct disorder and obsessive–compulsive disorder symptoms were assessed through parental report by means of the CBRS (Conners et al., 2011) using T‐scores on a 0–3 scale. Parental report was chosen over self‐report because more parents than adoptees completed these measures. However, parent‐ and adoptee self‐report of emotional problems were similarly elevated in young‐adulthood in the High Deprivation group (see Sonuga‐Barke et al., 2017). *T*‐scores falling below 60 suggest the absence of concerns.

#### Mental health service use

Young‐adult adoptees reported mental health service use since mid‐adolescence during an interview. A score of 1 (0–1 scale) indicated at least a two‐session contact with a mental health professional that resulted in either psychiatric diagnosis or the conclusion that there was a clinically significant disorder.

#### Life satisfaction

Participants rated this using the five‐item Satisfaction with Life Scale (SWLS; Diener, Emmons, Larsen, & Griffin, 1985; from 1 – strongly disagree to 5 – strongly agree).

#### Self‐esteem

The Rosenberg self‐esteem scale (Rosenberg, 1965), containing 10 self‐rated items measuring positive and negative feelings ranging from ‘strongly agree’ (4) to ‘strongly disagree’ (1). Total scores between 15 and 25 are within the normal range.

### Education, employment and daily functioning in adulthood

#### Education

Adoptees reported the highest level of education achieved: 0 (0–1 scale) denoted low achievement (GCSE or lower).

#### Academic achievement

Parents reported Maths and English difficulties using the CBRS (Conners et al., 2011). *T*‐scores falling below 60 suggested no concerns.

#### Employment

Adoptees reported their current employment status (Angold et al., 1999 **)**. A score of 0 denoted not “in employment, formally enrolled in full‐time education, or training”.

#### Disability

Parents reported whether adoptees were registered disabled: 0 denoted ‘not registered disabled’ on a 0 to 1 scale (Angold et al., 1999).

#### Daily functioning

The RAPFA interview (Hill et al., 2008) with the young people assessed day‐to‐day functioning in relation to finances, household tasks, and daily routines: 0 denoted ‘manage independently’ and 1 ‘unable to manage or manage with support’.

### Relationships measured in adulthood

#### Romantic relationships

Adoptees reported whether they had ever been in a romantic relationship (Angold et al., 1999): 0 (0–1 scale) indicated ‘never in a romantic relationship’.

#### Offspring

Adoptees reported if they had had a child. A score of 0 (0–1 scale) indicated ‘never had a child’ (Angold et al., 1999).

#### Relationship with parents

Adoptees completed the Inventory of Parent and Peer Attachment (IPPA; Armsden & Greenberg, 1987): a 25‐item self‐report questionnaire that taps into the construct of *degree of mutual trust* (10 items), *quality of communication* (9 items) and *extent of anger and alienation* (6 items). The IPPA uses a 5‐point Likert scale (1 = almost never true to 5 = almost always true).

### Statistical analysis

To answer questions 1a (*Does the Childhood QA+ group display persisting symptoms in all three autism core domains (social reciprocity, communication, and repetitive and stereotyped behaviours) across adolescence through to adulthood?*) and 1b (*Does the Childhood QA+ group display elevated trajectories of other DSPs into adulthood i.e. ADHD, DSE and cognitive impairment*), the intercept and linear and quadratic slopes of growth trajectories across ages 6, 11, 15 years and young‐adulthood were estimated using Latent Growth Models (LGM), which were subsequently included as dependent variables in one‐way ANOVAs with group (*Low/No Deprivation* vs. *Childhood QA−* vs. *Childhood QA+*) as the independent variable. Post‐hoc testing was performed, with Bonferroni correction, to determine the specificity of significant group effects. A cross‐sectional comparison of deprivation specific problems at young‐adult follow‐up with group as the independent variable was also performed. To answer question 1c (*Does the Childhood QA+ group experience poor mental health, impaired daily functioning, reduced life satisfaction and self‐esteem in adulthood*), we conducted group comparisons using ANOVA for continuous outcomes and $\chi^{2}$ for binary outcomes. We used a similar approach to address the remaining questions. Both involved using Multiple Linear Regression. For question 2 (*Is the association between childhood QA and adult functioning and mental health independent of the effects of the other deprivation‐specific neurodevelopmental problems – DSE, ADHD and cognitive impairment, expressed from childhood to adolescence?*), we included mean childhood to adolescence ADHD, DSE, and IQ scores and QA membership (*Childhood QA−* and *Childhood QA+*) as predictors, and adult mental health and functional impairment as dependent variables. For question 3 (*Which autism symptom domain (social reciprocity, communication, and/or repetitive and stereotyped behaviours) is most strongly associated with the negative impact of QA on adult outcomes (i.e. mental health and functional impairment)?*), we included mean childhood‐to‐adolescence SCQ domain scores as predictors, and adult mental health and functional impairment as dependent variables. Because we were specifically interested in the predictive effects related to deprivation, we restricted our analyses to those who experienced more than 6 months of institutional deprivation.

Prior to these regression analyses, two sets of principal component analysis (PCA) reduced the number of dependent variables measuring mental health problems and functional impairment into two separate individual scores for mental health problems and functional impairment, respectively. The PCA analysis for the *mental health facto*r included parent‐rated CBRS T‐scores for major depressive disorder, manic episodes, generalised anxiety disorder, social phobia and obsessive–compulsive disorder (Bartlett's test of sphericity: χ^2^ (10) = 265.924, *p* < .001; Kaiser–Meyer–Olkin (KMO) = .82; Total variance explained = 74.21%). The second PCA for the *functional impairment* factor included self‐reported RAPFA scores for daily functioning skills, household tasks, finances management, living independently, education, and employment status (Bartlett's test: $\chi^{2}$(15) = 220.646, *p* < .001; KMO = .81; Total variance explained = 56.85%). For both regression models, single predictor variables (SCQ scores, ADHD, DSE and IQ) were created by averaging outcomes at ages 6, 11 and 15 years. All tests, except LGM, were run using SPSS version 26. For LGM we used MPlus version 8.7.

## Results

In adulthood, *Childhood QA+* and *Childhood QA−* did not differ in terms of sex ratio, age, length of time in institutions, SES or whether parents are still living together. Both groups had more girls, were older and had spent more time in institutions than the *Low/No Deprivation* group (see Table 1).

**Table 1:** Comparison of adoptees' background characteristics, adult social functioning, mental health & wellbeing, and relationships by group

	Low/No Dep (*n* = 116)	High Dep (Rom > 6)		Main effect	Post hoc comparisons
		Childhood QA− (*n* = 75)	Childhood QA+ (*n* = 26)		
*Background characteristics*					
Age – mean years (SD)	23.5 (0.7)	24.5 (0.7)	24.3 (0.7)	***F*(2, 132) = 30.09, *p* < .001**	Low Dep < QA+ & QA−
Sex (% female)	42.2	57.3	65.4	**χ** ^**2**^ **(2) = 6.86, *p* = .032**	Low Dep < QA+ & QA−
Deprivation in months, mean (SD)	2.1 (1.9)	19.3 (9.6)	18.5 (10.5)	***F*(2, 162) = 92.99, *p* < .001**	Low Dep < QA+ & QA−
SES (% low)	11.4	18.8	20.8	χ^2^ (2) = 2.39, *p* = .302	–
Adoptive parents (% still together)	78.7	65.9	80	χ^2^ (2) = 2.85, *p* = .241	–
*Social functioning*					
Low education (GCSE or less) %	26.1	32.1	59.1	**χ** ^**2**^ **(2) = 8.72, *p* = .013**	QA+ > QA− & Low Dep
Unemployed %	11.2	26.4	59.1	**χ** ^**2**^ **(2) = 23.79, *p* < .001**	QA+ > QA− > Low Dep
Registered disabled %	1.1	5.7	43.5	**χ** ^**2**^ **(2) = 43.03, *p* < .001**	QA+ > QA− & Low Dep
Never lived independently %	24.7	22.6	50	**χ** ^**2**^ **(2) = 6.57, *p* = .037**	QA+ > QA− & Low Dep
Difficulty handing finances %	22.2	43.4	83.3	**χ** ^**2**^ **(2) = 11.67, *p* = .003**	QA+ > QA− > Low Dep
Difficulty household tasks %	12.2	30.2	75	**χ** ^**2**^ **(2) = 38.35, *p* < .001**	QA+ > QA− > Low Dep
Difficulty daily routines %	15.6	28.3	66.7	**χ** ^**2**^ **(2) = 25.22, *p* < .001**	QA+ < QA− & Low Dep
*Mental health and wellbeing*					
Mental health service use %	14.9	38.1	68.4	**χ** ^**2**^ **(2) = 24.67, *p* < .001**	QA+ > QA− > Low Dep
Life satisfaction, mean (SD)	16.9 (5.4)	16.9 (5.6)	15.8 (5.3)	*F*(2, 116) = .23, *p* = .794	–
Self‐esteem, mean (SD)	17.3 (3.3)	16.0 (3.3)	15.4 (3.7)	*F*(2, 117) = 3.17, *p* = .056	–
*Relationships*					
Ever in a romantic relationship %	87.5	86.3	57.1	**χ** ^**2**^ **(2) = 11.67, *p* = .003**	QA+ < QA− & Low Dep
Ever had a child %	14.6	11.3	18.2	χ^2^ (2) = .66, *p* = .718	–
Relationship with mother, mean (SD)	98.0 (19.1)	92.3 (26.4)	104.0 (22.6)	*F*(2, 114) = 1.57, *p* = .213	–
Relationship with father, mean (SD)	96.7 (20.5)	97.3 (22.4)	101.6 (14.9)	*F*(2, 107) = .28, *p* = .753	–

SD, Standard deviation. SES, Social‐Economic Status. Figures in bold refer to significant effect (i.e. *p* < .05). Low/No Dep, Low/No Deprivation. High Dep, High Deprivation. For full analysis output see Appendix S3 – Table S5.

### Is QA associated with a persisting pattern of symptoms?

**Figure 1:**
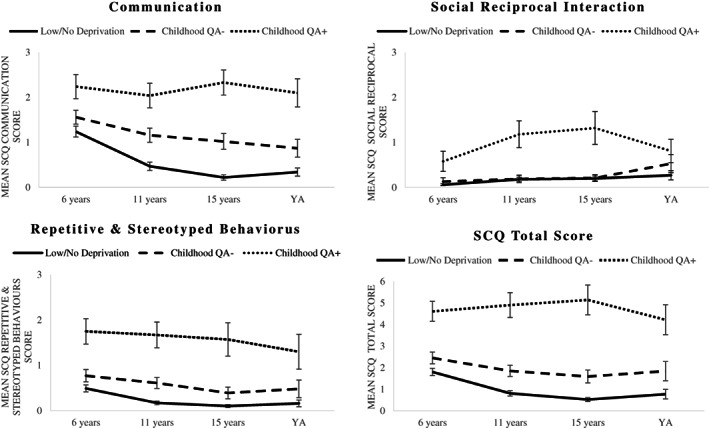
Developmental trajectories for autism symptoms. Error bars represent standard error of the mean. SCQ, Social Communication Questionnaire; YA, young adulthood (average age 23 years)

**Figure 2:**
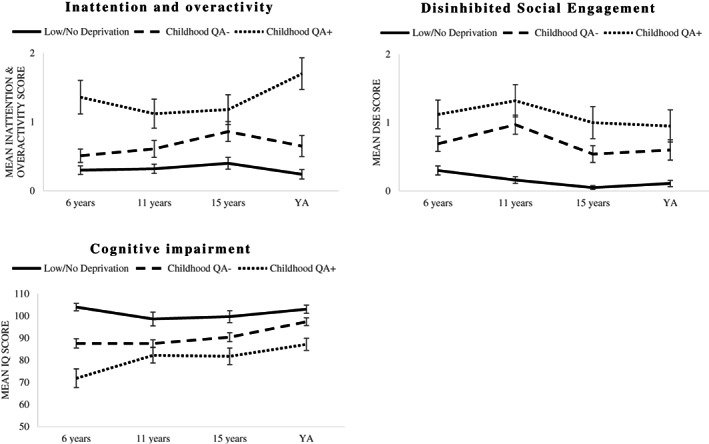
Developmental trajectories for deprivation specific problems symptoms. Error bars represent standard error of the mean. YA, young adulthood (average age 23 years). DSE, symptoms of disinhibited social engagement disorder

The intercept value was greater for *Childhood QA+* than *Childhood QA−* and *Low/No Deprivation* groups with *Childhood QA+* being associated with significantly higher levels of symptoms on all autism subscales, ADHD, DSE and lower IQ (Figures 1 and 2; Table 2). The groups also differed in terms of the slopes with a relative increase in communication difficulties and a relative decrease in repetitive and stereotyped behaviours for the *Childhood QA+* group compared to the other two groups. An examination of the slope values suggested that both *Childhood QA+* and *Childhood QA−* groups saw a relative worsening of DSE compared to the *Low/No Deprivation* group. Models for all variables failed to converge when the quadratic term was added to them. In young adulthood the difference between the groups was still significant for communication, repetitive and stereotyped behaviours, ADHD, DSE and lower IQ (Appendix S3 – Table S4).

**Table 2:** Comparison of growth trajectories for SCQ domains and deprivation specific problems by group

	Low/No Dep (*n* = 116)	High Dep (Rom >6)		Main effect	Post hoc comparisons
		Childhood QA− (*n* = 74)	Childhood QA+ (*n* = 26)		
*Intercept*					
Autism screening – SCQ, mean (*SD*)					
Social reciprocal interaction	0.07 (0.23)	0.13 (0.66)	0.63 (1.10)	***F*(2, 213) = 10.39, *p* < .001**	QA+ > QA− & Low/No Dep
Communication	1.04 (0.60)	1.40 (0.69)	1.86 (0.73)	***F*(2, 213) = 19.07, *p* < .001**	QA+ > QA− & Low/No Dep
Repetitive and stereotyped behaviours	0.40 (0.40)	0.70 (0.74)	1.58 (0.98)	***F*(2, 213) = 38.74, *p* < .001**	QA+ > QA− > Low/No Dep
Deprivation specific problems, mean (*SD*)					
Attention deficit/hyperactivity disorder	0.29 (0.65)	0.51 (0.83)	1.30 (1.05)	***F*(2, 213) = 18.27, *p* < .001**	QA+ > QA− & Low/No Dep
Disinhibited social engagement	0.39 (0.19)	0.59 (0.35)	0.79 (0.33)	***F*(2, 214) = 29.12, *p* < .001**	QA+ > QA− > Low/No Dep
IQ	101.23 (14.87)	88.09 (1.49)	79.93 (14.83)	***F*(2, 208) = 33.52, *p* < .001**	QA+ < QA− < Low/No Dep
*Slope*					
Autism screening – SCQ, mean (SD)					
Social reciprocal interaction	0.01 (0.03)	0.02 (0.05)	0.03 (0.07)	*F*(2, 213) = .89, *p* = .410	‐
Communication	−0.04 (0.06)	−0.02 (0.08)	0.03 (0.11)	***F*(2, 213) = 10.60, *p* < .001**	QA+ > QA− & Low/No Dep
Repetitive and stereotyped behaviours	−0.008 (0.01)	−0.017 (0.02)	−0.042 (0.03)	***F*(2, 213) = 37.07, *p* < .001**	QA+ < QA− < Low/No Dep
Deprivation specific problems, mean (*SD*)					
Attention deficit/hyperactivity disorder	0.029 (0.027)	0.031 (0.031)	−0.005 (0.05)	*F*(2, 213) = 2.21, *p* = .112	‐
Disinhibited social engagement	−0.02 (0.04)	−0.002 (0.08)	0.02 (0.11)	***F*(2, 214) = 4.77, *p* = .009**	QA+ > Low/No Dep
IQ	0.06 (0.36)	0.34 (0.35)	0.41 (0.41)	***F*(2, 208) = 17.81, *p* < .001**	Low/No Dep < QA+ & QA−

SD, standard deviation. SCQ, Social Communication Questionnaire. IQ, intelligence quotient. Figures in **bold** refer to significant effect. Low/No Dep, Low/No Deprivation. High Dep, High Deprivation. For full analysis output see Appendix S3 – Table S6.

### Is QA associated with more negative adult outcomes?

*Childhood QA+* was associated with impaired functioning and psychopathology (see Table 1). Eight times as many *Childhood QA+* adults were registered disabled than their *Childhood QA−* counterparts. They were twice as likely never to have lived independently and reported higher functional impairment across all domains (i.e. routines, household tasks and finances). They had significantly lower levels of educational attainment and substantially higher rates of unemployment. They were less likely to have had a romantic relationship (see Table 1). Individuals in the *Childhood QA+* group were twice as likely to have used mental health services as their *Childhood QA−* counterparts. They displayed elevated scores across psychopathology categories compared to *Childhood QA−* (Table 3). Interestingly, the groups did not differ in the quality of their relationships with family or in life satisfaction and self‐esteem (see Table 1).

**Table 3:** Comparison of young adult adoptees' mental health and academic problems by group

	Low/No Dep (*n* = 116)	High Dep (Rom >6)		Main effect	Post hoc comparisons
		Childhood QA− (*n* = 75)	Childhood QA+ (*n* = 26)		
*Mood and behavioural disorders*, mean (*SD*)					
Conduct disorder	48.6 (8.8)	53.0 (11.8)	59.3 (14.9)	***F*(2, 140) = 8.533, *p* < .001**	QA+ > Low/No Dep
Oppositional defiant disorder	50.6 (12.7)	57.8 (17.5)	69.9 (15.5)	***F*(2, 140) = 14.516, *p* < .001**	QA+ > QA− > Low/No Dep
Major depressive episode	56.9 (17.2)	64.5 (21.1)	77.3 (14.8)	***F*(2, 140) = 10.573, *p* < .001**	QA+ > QA− & Low/No Dep
Manic episode	52.1 (12.0)	60.3 (20.2)	74.7 (17.6)	***F*(2, 140) = 17.335, *p* < .001**	QA+ > QA− > Low/No Dep
Generalised anxiety disorder	57.3 (15.6)	64.9 (19.7)	79.6 (13.8)	***F*(2, 140) = 14.655, *p* < .001**	QA+ > QA− & Low/No Dep
Social phobia	51.9 (12.0)	55.4 (15.0)	68.3 (18.2)	***F*(2, 140) = 11.175, *p* < .001**	QA+ > QA− & Low/No Dep
Obsessive–compulsive disorder	51.6 (11.8)	58.7 (18.9)	77.1 (19.9)	***F*(2, 140) = 21.072, *p* < .001**	QA+ > QA− & Low/No Dep
*Academic problems*, mean (*SD*)					
Language	52.2 (14.0)	59.6 (16.6)	75.8 (15.5)	***F*(2, 139) = 20.440, *p* < .001**	QA+ > QA− > Low/No Dep
Math	58.0 (17.8)	66.3 (18.8)	78.2 (16.0)	***F*(2, 140) = 11.077, *p* < .001**	QA+ > QA− > Low/No Dep

SD, standard deviation. Figures in bold refer to significant effect (i.e., *p* < .05). Low/No Dep, Low/No Deprivation. High Dep, High Deprivation. For full analysis output see Appendix S3 – Table S7.

### Is QA associated with worse outcomes after controlling for other deprivation specific problems?

The overall regression models for both *mental health problems* (*F*(4, 53) = 5.75, *p* < .001, $R^{2}$ = .25) *and functional impairment* (*F*(4, 62) = 13.56, *p* < .001, $R^{2}$ = .43) were significant. QA Group membership (*t* = 2.25, *p* = .028) and ADHD (*t* = 2.06, *p* = .044) were significant independent predictors for the former; while IQ (*t* = −3.78, *p* < .001) and QA Group membership (*t* = 2.77, *p* = .008), were significant for the latter.

### Which autism sub‐domains drive adult problems?

Regression models were significant (*F* ^mental health^ (3, 60) = 5.00, *p* = .004, $R^{2}$ = .17; *F* ^impairment^ (3, 70) = 4.55, *p* = .006, $R^{2}$ = .13), with communication making a significant independent prediction (*t* = 2.83, *p* = .006) to the former, and communication (*t* = 2.21, *p* = .03) and social reciprocity (*t* = 2.19, *p* = .03) to the latter.

## Discussion

Rutter's discovery that a considerable proportion of children exposed to extreme, global institutional deprivation showed increased rates of autism symptoms raised challenging questions about both the causes of autism and the impact of early maltreatment (Rutter et al., 2007). We present the first detailed and comprehensive longitudinal analysis of the adult outcomes of these young people. There were a number of notable findings. First, there is strong continuity of autism symptoms into adulthood – despite the adoptees being raised in well‐functioning families for over 20 years. This was also seen for deprivation‐related ADHD and DSE (Kennedy et al., 2016, 2017). It is consistent with the notion that exposure to institutional deprivation during early development can produce deep‐seated neuro‐developmental alterations, which, after a period of rapid catch‐up in the years soon after adoption, are largely insensitive to later environmental enrichment.

Second, QA manifests across all three autism domains – although there are subtle differences in developmental trajectories through to adulthood: Communication problems worsened somewhat, while repetitive and stereotyped behaviours, although persistent, improved to a certain degree. Worsening communication problems is especially notable since, according to Rutter et al. (1999), a relative lack of communication problems was a distinctive feature of QA in the early years. Regarding repetitive and stereotyped behaviours, as previously mentioned, QA had a distinctive pattern limited largely to intense circumscribed interests and/or abnormal preoccupations (Rutter et al., 1999). Although the adapted repetitive and stereotyped behaviours scale used here did include some ritualistic and stereotype behaviours, these were not sufficient for a detailed analysis of this issue. Previous longitudinal studies of the adult outcomes of idiopathic autism suggest that, where symptom improvements occur, it is across all domains, including communication problems (Howlin, Moss, Savage, & Rutter, 2013), highlighting a possible difference between deprivation and non‐deprivation‐related autism. Finally, interestingly, elevation of autism symptoms was not limited to the *Childhood QA+* group: those in the *Childhood QA−* group also had elevated levels of autism symptoms compared to the *Low/No Deprivation* group. This supports the notion that QA is more a continuum than a category and we should consider sub‐threshold symptoms when looking at clinical profiles.

Third, there was substantial overlap between QA and the other deprivation‐specific problems (ADHD, DSE and cognitive impairment) in adulthood similar to that seen in adolescence (see Kreppner et al., 2010). While ADHD (Panagiotidi, Overton, & Stafford, 2019) and cognitive impairment (Howlin, Savage, Moss, Tempier, & Rutter, 2014) commonly coexist with autism in non‐deprived adult samples, DSE appears particularly characteristic of adult QA. This conforms to Rutter's initial observation. Some studies have also observed a combination of DSE and autism in children with a history of abuse and/or neglect (Mayes, Calhoun, Waschbusch, & Baweja, 2017; McCullough, Stedmon, & Dallos, 2014; Pritchett, Pritchett, Marshall, Davidson, & Minnis, 2013; Sadiq et al., 2012). The persistent co‐occurrence of DSE and autism, as well as ADHD, in adults with a history of childhood maltreatment raises questions about the causes driving this overlap and the possibility that together they may constitute the core of what might be called neuro‐developmental deprivation ‘syndrome’.

Fourth, QA is associated with a profound set of challenges in adulthood as manifested by the high levels of mental health problems and functional impairments observed – effects not explained by overlapping deprivation‐specific problems. These findings highlight the functional impact of QA and the associated clinical needs of this group. Recent studies of the adult outcome of individuals with a childhood diagnosis of idiopathic autism show a similar picture with poor outcomes in employment and education (Toft et al., 2021), independent living (Billstedt, Gillberg, & Gillberg, 2011) and mental health (Lever & Geurts, 2016). A direct comparison with a cohort of non‐deprived autistic individuals is needed to calibrate the relative severity of these outcomes.

Lastly, through all their life challenges, relationships with parents remained on average good, although the ability to form romantic relationships seemed to be negatively impacted. The QA adoptees' sense of life satisfaction and self‐worth was strong. This contrasts with the findings from studies of adults with idiopathic autism whose outcomes are typically negatively impacted (Nguyen, Ownsworth, Nicol, & Zimmerman, 2020; Schmidt et al., 2015; Soares et al., 2021). Possible explanations for these differences requiring further exploration include special qualities of ERA families including parenting (Castle, Beckett, Rutter, & Sonuga‐Barke, 2010) and resilience (Shmotkin, 2005) and/or cohort differences in terms of IQ, given it has been suggested that autistic individuals with higher IQ have better awareness of their limitations and deficits (Huang et al., 2017).

The high rates and strong persistence of autism in the ERA study appear to support a causal role for institutional deprivation in QA. Other explanations of the statistical link though need to be considered. It is possible for instance that there were extremely high rates of idiopathic autism risk in the families placing their children in the institutions. However, although poverty‐related pre‐ and perinatal autism risk factors were likely common in these families, based on current estimates of effect sizes of such risks in the literature, they could not possibly account for the rates of QA seen in ERA (Mayes et al., 2017). Finally, the distinctive features of the QA group would suggest that this was not typical autism. It is also possible that families selectively placed children most at risk for autism in the institutions. However, this is highly improbable given that placements were typically made within the first months of life, where signs of early autism would be extremely difficult to spot.

Despite the methodological strengths of the ERA study there are some limitations. First, there was no comprehensive clinical assessment of autism in adulthood and so diagnostic status could not be established. Second, in order to ensure comparability across ages the assessment of autism symptoms using the SCQ relied on a restricted range of items within each domain. This meant that the assessment used at a particular age may have missed important developmentally relevant characteristics of QA. Third, there was considerable attrition between the adolescence and adult waves which reduced statistical power. However, there was sufficient power to show QA‐specific differences across most outcomes where effect sizes were moderate. The possibility that we missed some less pronounced but still important difference cannot be ruled out.

Our findings have clinical implications. First, since deprivation‐related autism is associated with poor outcomes in adulthood, it is imperative that autistic adoptees who have suffered from early‐in‐life deprivation receive adequate specialist support during the adolescence‐to‐adulthood transition (Anderson, Newlove‐Delgado, & Ford, 2022). Second, measuring exposure to early deprivation in children with autism during clinical assessment seems important given the differences between QA and idiopathic autism. Third, we should reflect on whether QA still stands as the right term to refer to autism symptoms following early deprivation. Using the term might lead some clinicians to see the associated problems as idiosyncratic and transitory, limiting adoptees access to evidence‐based interventions for autism (Mayes, Breaux, Calhoun, & Whitmore, 2019). Fourth, the question of whether other forms of maltreatment can create risk for QA needs further consideration. QA has been observed in other cohorts of children exposed to less severe institutional deprivation (i.e. Bucharest Early Intervention Project; Levin, Fox, Zeanah, & Nelson, 2015), and in children adopted after early disrupted care and maltreatment (Green, Leadbitter, Kay, & Sharma, 2016).

In summary, QA is persistent and complex and impacts the adult lives of the individual it effects in profound ways. Clinical services should not consider this form of autism as transitory or benign.

## Supporting information


**Appendix S1.** Autism screening comparison between QA+ and QQA across age waves.
**Appendix S2.** Selection of Social Communication Questionnaire (SCQ) items.
**Appendix S3.** Supplementary statistical analyses.
**Table S1.** Autism screening comparison between QA+ and QQA adoptees at age 6, 11, 15 and Young Adulthood.
**Table S2.** Items by autism symptom domain by waves.
**Table S3.** Excluded items and reason for exclusion.
**Table S4.** Comparison of autism symptoms and other deprivation specific problems in YA between groups.
**Table S5.** Post hoc comparisons of adoptees' background characteristics, adult social functioning, mental health and wellbeing, and relationships.
**Table S6.** Post hoc comparisons of adoptees' rates of growth.
**Table S7.** Post hoc comparison of young adult adoptees' mental health symptoms and academic problems.
