# Supplementary material for: The adult outcome of childhood quasi‐autism arising following extreme institutional deprivation
Source: J Child Psychol Psychiatry. 2023 Feb 13;64(9):1292–302. doi: 10.1111/jcpp.13767 (PMC10476691; doi:10.1111/jcpp.13767)
Supplement: Supplementary file 1 — Appendix S1. Autism screening comparison between QA+ and QQA across age waves. Appendix S2. Selection of Social Communication Questionnaire (SCQ) items. Appendix S3. Supplementary statistical analyses. Table S1. Autism screening comparison between QA+ and QQA adoptees at age 6, 11, 15 and Young Adulthood. Table S2. Items by autism symptom domain by waves. Table S3. Excluded items and reason for exclusion. Table S4. Comparison of autism symptoms and other deprivation specific problems in YA between groups. Table S5. Post hoc comparisons of adoptees' background characteristics, adult social functioning, mental health and wellbeing, and relationships. Table S6. Post hoc comparisons of adoptees' rates of growth. Table S7. Post hoc comparison of young adult adoptees' mental health symptoms and academic problems. [file JCPP-64-1292-s001.docx]

**Supporting Information**

**The Adult Outcome of Childhood Quasi-Autism arising following Extreme Institutional Deprivation Contents**

Maria Rodriguez Perez, Mark Kennedy, Edward D Barker, Jana Kreppner, Mireia Solerdelcoll and Edmund Sonuga-Barke

**Contents**

Appendix S1. Autism screening comparison between QA+ and QQA across age waves

Appendix S2. Selection of Social Communication Questionnaire (SCQ) items

Appendix S3. Supplementary statistical analyses

**Appendix S1. Autism screening comparison between childhood QA+ and childhood QQA across age waves**

**Table S1. Autism Screening comparison between QA+ and QQA adoptees at age 6, 11, 15 and Young Adulthood**

|  | **6 years** | |  | **11 years** | | |  |
| --- | --- | --- | --- | --- | --- | --- | --- |
|  | Childhood QA+ (n=15) | Childhood QQA (n=11) | QA+ *vs* QQA | Childhood  QA+ (n=15) | Childhood  QQA (n=11) | | QA+ *vs* QQA |
| **Autism Screening - SCQ, mean (SD)** |  |  |  |  |  |  |  |
| Social Reciprocal Interaction | 0.93 (1.38) | 0.09 (.30) | **t= 2.280, p= .037** | 1.58 (1.65) | .63 (1.20) | | t= 1.609, p= .121 |
| Communication | 2.14 (1.17) | 2.35 (1.50) | t= -.414, p= .683 | 1.73 (1.01) | 2.45 (1.75) | | t= -1.222, p= .241 |
| Repetitive & Stereotyped behaviours | 2.05 (1.31) | 1.30 (1.49) | t= 1.326, p= .198 | 1.70 (.99) | 1.63 (1.96) | | t= .099, p= .923 |
| SCQ Total Score | 5.01 (2.48) | 4.02 (2.04) | t= 1.039, p= .310 | 5.03 (2.27) | 4.73 (3.80) | | t= .254, p= .802 |
|  | **15 years** | |  |  | **YA** |  |  |
|  | Childhood QA+ (n=12) | Childhood QQA (n=10) | QA+ *vs* QQA | Childhood  QA+ (n=11) | | Childhood  QQA (n=9) | QA+ *vs* QQA |
| **Autism Screening - SCQ, mean (SD)** |  |  |  |  |  |  |  |
| Social Reciprocal Interaction | 2.00 (1.95) | 0.50 (.85) | **t= 2.250, p= .029** | 1.11 (1.37) | | 0.44 (.73) | t= 1.313, p= .206 |
| Communication | 2.27 (1.40) | 2.40 (1.26) | t= -.225, p= .825 | 2.27 (1.25) | | 1.89 (1.61) | t= .599, p= .557 |
| Repetitive & Stereotyped behaviours | 1.95 (1.77) | 1.11 (1.45) | t= 1.148, p= .266 | 1.64 (1.74) | | 0.89 (1.69) | t= .965, p= .347 |
| SCQ Total Score | 6.11 (3.57) | 3.99 (2.48) | t= .170, p= .129 | 5.04 (3.67) | | 3.22 (1.98) | t= 1.334, p= .199 |

*Note*. Childhood QA+, adoptees identified with QA symptoms during childhood assessment. Childhood QQA, adoptees identified with queried QA symptoms during childhood assessment. SCQ, Social Communication Questionnaire. SD, Standard Deviation. YA, Young adult (average age 23 years ). Figures in **bold** refer to significant effect (i.e., p < .05).

**Appendix S2. Items used for autism symptoms domains across age waves**

Preamble: To ensure its developmental appropriateness in young adulthood, items from the full SCQ were dropped on the basis of their distribution in the combined UK and Rom<6 group because they were (i) too commonly endorsed in young adulthood and/or (ii) showed a substantial increase between age 6 years and young adulthood - patterns inconsistent with items being considered markers of a serious/rare neuro-developmental condition. Full item descriptions are withheld due to copyright.

**Table S2. Items by autism symptom domain by waves**

| ***Autism Spectrum Disorder*** |  |  |  |
| --- | --- | --- | --- |
| **Age 6** | **Age 11** | **Age 15** | **YA - Parent report** |
| **Social Reciprocal Interaction** | As age 6 | As age 6 | As age 6 |
| 17. Smiles back |  |  |  |
| 21. Attempts to comfort |  |  |  |
| 23. Normal range of facial expressions |  |  |  |
| 24. Appropriate facial expressions |  |  |  |
| 28. Responds positively to others |  |  |  |
| **Communication** |  |  |  |
| 9. Odd speech |  |  |  |
| 10. To-and-fro conversation |  |  |  |
| 11. Socially appropriate |  |  |  |
| 12. Difficulties with pronouns |  |  |  |
| 13. Uses made up words/phrases |  |  |  |
| **Repetitive and Stereotyped Behaviours** |  |  |  |
| 33. Odds interest |  |  |  |
| 34. Interested in parts of objects. |  |  |  |
| 35. Ritualised behaviour |  |  |  |
| 36. Unusual interest in smell etc. of things or people |  |  |  |
| 38. Odd mannerisms/movement |  |  |  |

*Note*. Item description from the SCQ is withheld due to copyright. YA, Young adulthood.

**References:**

Rutter M, Bailey A, Lord C. SCQ. The social communication questionnaire. Torrance: Western Psychological Services, 2003

**Table S3. Excluded items and reason for exclusion**

| **Item** | **Commonly endorsed in young adulthood** | **Increased between age 6 and young adulthood** |
| --- | --- | --- |
| 4. Spontaneously point at things | X | X |
| 6. Nod head for yes | X | X |
| 7.Shake head for no | X | X |
| 8. Talk to be friendly | X |  |
| 14. Repetitive speech | X |  |
| 16. Eye contact | X |  |
| 18. Show objects of interest | X |  |
| 19. Share interest | X | X |
| 20. Wants others to join in | X | X |
| 22. Speech and gestures to get attention | X |  |
| 26. Plays make believe games | X | X |
| 27. Interest in peers |  |  |
| 29. Plays imaginative games | X | X |
| 30. Special interests | X |  |
| 31. Joins in with games | X |  |
| 32. Any particular friends | X |  |
| 39. Stereotyped behaviours | X |  |

*Note*. The following items were not included as they do not constitute part of any subscale; 15. Attends to you, 37. Special objects, 40. Selfharm/ head banging.

**Appendix S3. Supplementary statistical analyses**

**Table S4. Comparison of Autism symptoms and other Deprivation Specific Problems in YA between groups.**

|  | **Low/No Dep(n=116)** | **HiDep (Rom > 6)** | | **Main effect** | **Post hoc comparisons** | Low/No Dep *vs* Childhood QA+ | Childhood QA- *vs* Childhood QA+ | |
| --- | --- | --- | --- | --- | --- | --- | --- | --- |
|  |  | Childhood | Childhood |  |  |  |  |  |
|  |  | QA- (n=75) | QA+ (n=26) |  |  |  |  |  |
| ***Autism screening in YA, mean (SD)*** |  |  |  |  |  |  |  |  |
| Social Reciprocal Interaction | 0.3(0.9) | 0.5(1.3) | 0.8(1.2) | F (2,139) = 2.159, p = .119 | - | t = -1.895, *p* =.070 | t = -.805, *p* =.424 | |
| Communication | 0.3(0.8) | 0.9(1.3) | 2.1(1.4) | **F (2,139) = 21.598, p < .001** | QA+ > QA- > Low/No Dep | **t = -5.274, *p* <.001** | **t = -3.366, *p* =.001** | |
| Repetitive and Stereotyped Behaviours | 0.2(0.7) | 0.5(1.3) | 1.3(1.7) | **F (2,139) = 9.347, p < .001** | QA+ > QA- & Low/No Dep | **t = -2.904, *p* =.009** | t = -1.914, *p* =.066 | |
| SCQ Total Score | 0.8(2.0) | 1.9(2.9) | 4.2(3.1) | **F (2,139) = 15.716, p < .001** | QA+ > QA- & Low/No Dep | **t = -4.689, *p* <.001** | **t = -2.857, *p* =.006** | |
| ***Other Deprivation Specific Problems in YA, mean (SD)*** |  |  |  |  |  |  |  | |
| ADHD | 50.47 (10.59) | 57.0 (14.76) | 71.92 (13.44) | **F (2,140) = 24.544, p < .001** | QA+ > QA- > Low/No Dep | **t = -7.666, *p* <.001** | **t = -3.839, *p* <.001** | |
| DSE | 0.11 (.42) | 0.60 (0.98) | 0.95 (1.05) | **F (2,142) = 13.823, p < .001** | QA+ > Low/No Dep | **t = -3.512, *p* =.002** | t = -1.274, *p* =.208 | |
| IQ | 102.98 (16.19) | 97.60 (11.23) | 87.13 (10.59) | **F (2,125) = 8.149, p < .001** | QA+ < QA- < Low/No Dep | **t = 3.631, p <.001** | **t = 3.337, p =.002** | |

YA, Young-Adulthood. SD, Standard deviation. SCQ, Social Communication Questionnaire. ADHD, Attention Deficit Hyperactivity Disorder. DSE, symptoms of Disinhibited Social Engagement Disorder. IQ, Intelligence quotient. Low/No Dep, Low/No Deprivation. High Dep, High Deprivation. Figures in **bold** refer to significant effect (i.e., p < .05).

**Table S5. Post hoc comparisons of adoptees’ background characteristics, adult social functioning, mental health & wellbeing, and relationships.**

|  | Low/No Dep *vs* Childhood QA+ | Childhood QA– vs Childhood QA+ |
| --- | --- | --- |
| **Background characteristics** |  |  |
| Age - mean years (SD) | **t = -4.475, *p* <.001** | **t = .838, *p* = .406** |
| Sex (% female) | **χ² (1) =4.573, *p* =.032** | χ² (1) =0.519, *p* =.471 |
| Deprivation in months | **t = -7.918, *p* <.001** | t = -.336, *p* = .738 |
| SES (% low) | - | - |
| Adoptive parents | - | - |
| Stressful life events | t = .893, *p* = .374 | **t = 1.996, *p* = .050** |
| Polygenic Risk Scores ASD | - | - |
| ***Social Functioning*** |  |  |
| Low education | **χ² (1) =8.681, p =.003** | **χ² (1) =4.728, p =.030** |
| Unemployed | **χ² (1) =24.591, p <.001** | **χ² (1) =7.205, p =.007** |
| Registered disability | **χ² (1) =37.019, p <.001** | **χ² (1) =16.178, p <.001** |
| Never lived independently | **χ² (1) =5.396, *p* =.020** | **χ² (1) =5.473, *p* =.019** |
| Difficulties handling finances | **χ² (1) =31.068, *p* <.001** | **χ² (1) =10.685, *p =*.001** |
| Difficulties with household tasks | **χ² (1) =39.369, *p* <.001** | **χ² (1) =13.453, *p* <.001** |
| Difficulties with daily routine | **χ² (1) =25.526, *p* <.001** | **χ² (1) =10.109, *p* <.001** |
| ***Mental health & wellbeing*** |  |  |
| Mental health service use | **χ² (1) =24.092, p <.001** | **χ² (1) =4.824, p =.028** |
| Life satisfaction | - | - |
| Self-Esteem | - | - |
| ***Relationships*** |  |  |
| Ever in a relationship | **χ² (1) =10.429, p =.003** | **χ² (1) =7.034, p =.012** |
| Ever had a child | - | - |
| Relationship with mother | - | - |
| Relationship with father | - | - |

SES, Social Economic Status. ASD, Autism Spectrum Disorder. Low/No Dep, Low/No Deprivation. Figures in **bold** refer to significant effect (i.e., p

**Table S6. Post hoc comparisons of adoptees’ rates of growth.**

|  | Low/No Deprivation vs Childhood QA+ | Childhood QA- vs Childhood QA+ |
| --- | --- | --- |
| **Intercept** |  |  |
| ***Autism Screening - SCQ*** |  |  |
| Social Reciprocal Interaction | **t = -2.574, p = .016, d'= -1.092** | **t = -2.171, p = .038, d'= -.624** |
| Communication | **t = -6.091, p < .001, d'= -1.322** | **t = -3.139, p = .002, d'= -.716** |
| Repetitive and Stereotyped Behaviours | **t = -6.041, p < .001, d'= -2.153** | **t = -4.191, p < .001, d'= -1.090** |
| ***Deprivation specific problems*** |  |  |
| Attention Deficit/Hyperactivity Disorder | **t = -4.716, p < .001, d'= -1.367** | **t = -3.870, p < .001, d'= -.882** |
| Disinhibited Social Engagement | **t = -5.895, p = .001, d'= -1.817** | **t = -2.475, p = .015, d'= -.563** |
| IQ | **t = 6.485, p < .001, d'= 1.431** | **t = 2.658, p = .009, d'= .618** |
| **Slope** |  |  |
| ***Autism Screening - SCQ*** |  |  |
| Social Reciprocal Interaction | t = -.935, p = .358, d'= -.323 | t = -.694, p = .492, d'= -.181 |
| Communication | **t = -3.447, p = .002, d'= -1.059** | **t = -2.495, p = .017, d'= -.642** |
| Repetitive and Stereotyped Behaviours | **t = 5.939, p < .001, d'= 2.112** | **t = 4.101, p < .001, d'= 1.062** |
| ***Deprivation specific problems*** |  |  |
| Attention Deficit/Hyperactivity Disorder | t = 1.446, p = .159, d'= .430 | t = 1.421, p = .165, d'= .395 |
| Disinhibited Social Engagement | t = -1.897, p = .069, d'= -.708 | t = -.916, p = .366, d'= -.239 |
| IQ | **t = -4.290, p < .001, d'= .947** | t = -.874, p = .385, d'= -.203 |

SCQ, Social Communication Questionnaire. IQ, Intelligence quotient. Low/No Dep, Low/No Deprivation. Figures in **bold** refer to significant effect (i.e., p < .05).

**Table S7. Post hoc comparison of young adult adoptees’ mental health symptoms and academic problems.**

|  | Low/No Dep *vs* Childhood QA+ | Childhood QA- *vs* Childhood QA+ |
| --- | --- | --- |
| ***Mood and Behavioural Disorders*** |  |  |
| Conduct Disorder | **t = -3.066, *p* = .006** | t = -1.808, *p* = .076 |
| Oppositional Defiant Disorder | **t = -5.805, *p* < .001** | **t = -2.651, *p* = .010** |
| Major Depressive Episode | **t = -4.852, *p* < .001** | **t = -2.757, *p* = .008** |
| Manic Episode | **t = -5.530, *p* < .001** | **t = -2.742, *p* = .008** |
| Generalised Anxiety Disorder | **t = -5.816, *p* < .001** | **t = -3.397, *p* = .001** |
| Social Phobia | **t = -3.842, *p* < .001** | **t = -2.971, *p* = .004** |
| Obsessive Compulsive Disorder | **t = -5.329, *p* < .001** | **t = -3.461, *p* < .001** |
| ***Academic problems*** |  |  |
| Language | **t = -6.636, *p* < .001** | **t = -3.686, *p* < .001** |
| Math | **t = -4.627, *p* < .001** | **t = -2.433, *p* = .018** |

Low/No Dep, Low/No Deprivation. Figures in **bold** refer to significant effect (i.e., p < .05).
